# Dynamics of ACTH-Mediated Regulation of Gene Transcription in ATC1 and ATC7 Adrenal *Zona Fasciculata* Cell Lines

**DOI:** 10.1210/en.2018-00840

**Published:** 2019-02-15

**Authors:** Georgina Hazell, George Horn, Stafford L Lightman, Francesca Spiga

**Affiliations:** Bristol Medical School: Translational Health Sciences, University of Bristol, Bristol, United Kingdom

## Abstract

We tested the hypothesis that mouse ATC1 and ATC7 cells, the first adrenocortical cell lines to exhibit a complete *zona fasciculata* (ZF) cell phenotype, respond to dynamic ACTH stimulation in a similar manner as the adrenal gland *in vivo*. Exploiting our previous *in vivo* observations that gene transcription within the steroidogenic pathway is dynamically regulated in response to a pulse of ACTH, we exposed ATC1 and ATC7 cells to various patterns of ACTH, including pulsatile and constant, and measured the transcriptional activation of this pathway. We show that pulses of ACTH administered to ATC7 cells can reliably stimulate a pulsatile pattern of transcriptional activity that is comparable to that observed in adrenal ZF cells *in vivo*. Hourly pulses of ACTH stimulate dynamic increases in CREB phosphorylation (pCREB) and transcription of genes involved in critical steps of steroidogenesis including signal transduction (*e.g.,* MRAP), cholesterol delivery (*e.g.,* StAR), and steroid biosynthesis (*e.g.,* CYP11A1), as well as those relating to transcriptional regulation of steroidogenic factors (*e.g.,* SF-1 and Nur-77). In contrast, constant ACTH stimulation results in a prolonged and exaggerated pCREB and steroidogenic gene transcriptional response. We also show that when a large dose of ACTH (100 nM) is applied after these treatment regimens, a significant increase in steroidogenic transcriptional responsiveness is achieved only in cells that have been exposed to pulsatile, rather than constant, ACTH. Our data support our *in vivo* observations that pulsatile ACTH is important for the optimal transcriptional responsiveness of the adrenal. Importantly, our data suggest that ATC7 cells respond to dynamic ACTH stimulation.

Glucocorticoids (principal endogenous glucocorticoids are cortisol in humans and corticosterone in mouse and rat) are steroid hormones that are important regulators of all mammalian physiological systems. Glucocorticoids are traditionally viewed as a stress hormone because of their release in response to acute and chronic stress [reviewed in (1, 2)], yet the actions of glucocorticoids are also pertinent to daily homeostatic control and are essential for developmental, metabolic, cardiovascular, immune, and neurobiological processes [reviewed in (3–7)]. Circulating glucocorticoids are released from the *zona fasciculata* (ZF) layer of the adrenal cortex mainly in response to anterior pituitary–derived ACTH. However, because of its lipophilic structure, glucocorticoids cannot be stored in the ZF cell. Therefore, ACTH stimulates a rapid nongenomic steroidogenic pathway that results in immediate *de novo* synthesis and release of glucocorticoids. This process is mediated by ACTH binding to MC2R (8) and activation of cAMP and, in turn protein kinase A (PKA) (8–10), leading to rapid phosphorylation of hormone-sensitive lipase (HSL) and steroidogenic acute regulatory protein (StAR), initiating a critical regulatory step in steroidogenesis: the mobilization and transfer of stored cholesterol to the inner mitochondrial membrane [reviewed in (11)]. Here cytochrome P450 side chain cleavage enzyme (gene name CYP11A1) sets off a series of enzymatic reactions that rapidly convert cholesterol to corticosterone [reviewed in (12)].

In addition to its rapid effects, ACTH also stimulates a delayed/genomic steroidogenic pathway, which modulates the CREB-dependent transcription of steroidogenic-related genes including MC2R, the MC2R accessory protein MRAP, StAR, and CYP11A1, presumably to prime the cell for the next surge in plasma ACTH. In addition to CREB, other transcription factors are also recruited to facilitate ACTH modulation of transcription of steroidogenic genes. Indeed, CREB-mediated transcription of StAR is increased by the activation of orphan nuclear receptor transcription factors steroidogenic factor-1 (SF-1) (13, 14) and Nur77 (15), encoded by the NR5A1 and NR4A1 genes, respectively, and negatively regulated by the atypical orphan nuclear receptor transcription factor DAX-1 (dosage-sensitive sex reversal-adrenal hypoplasia congenital critical region on X-chromosome, gene 1, encoded by the NR0B1 gene) (16). ACTH also modulates the expression of these transcription factors: ACTH increases the expression of the activators SF-1 and Nur77 but transiently downregulates the expression of the repressor DAX-1 (17, 18).

In mammals, ACTH and corticosterone are subject to a circadian pattern of release [reviewed in (19)] superimposed by discrete ultradian ACTH and corticosterone pulses that occur approximately every 60 minutes in rats (20–22) and 60 to 90 minutes in humans (23–25). We have shown that this episodic pattern is also translated at the level of the adrenal tissue as the phosphorylation of steroidogenic-related proteins and transcription of steroidogenic-related genes in the rat adrenal gland also follow an ultradian rhythm (26–28). There is evidence suggesting that changing the pattern or duration of ACTH stimulus can greatly disrupt steroidogenic-related dynamics and in turn corticosterone secretion. For example, we have shown that in rats with suppressed-endogenous HPA axis activity, hourly exogenous pulses of ACTH activate a pulsatile pattern of steroidogenic-related gene transcription and endogenous corticosterone secretion, whereas a constant ACTH infusion (at the same hourly dosage) does not stimulate a change in steroidogenic-related gene expression or corticosterone release (19, 27). This finding suggests that the pulsatile pattern of ACTH release is critical for optimal activation of the steroidogenic pathways and corticosterone synthesis and release in the adrenal gland. However, the mechanisms behind how the adrenal gland preferentially responds to a pulsatile pattern of ACTH are not fully understood. We have therefore followed up these studies into the dynamics of adrenal steroidogenesis by developing a model of pulsatile ACTH stimulation and conducting studies on ZF cells *in vitro.*

To date, only a few studies have investigated the effects of ACTH oscillations on ZF steroidogenic activity *in vitro,* and those that have were performed on isolated heterozygous adrenocortical cell populations (29, 30). This is in part because of the lack of a viable ZF cell line; in the past it has proved difficult to isolate the ZF from the surrounding cortical layers. Consequently, many studies of ACTH-mediated steroidogenesis have been conducted with a nonphenotypic human NCI-H295 cell line that shares the phenotypes of all three adrenal cortical layers (31), and only few have used the NCI-H295R subpopulations of NCI-H295 cells (H295R-S1, H295R-S2, H295R-S3), which have ZF-specific characteristics. However, the responses of these H295R strains to secretagogues, and therefore their functional aspects, vary significantly depending on the growth medium used (32).

Of the cell lines that have been isolated from the other layers and are a pure ZF population, few have proven to harbor a fully functioning ZF phonotype; for example, the mouse Y1 ZF cell line does not express cytochrome P450 21-hydroxylase and thus does not produce corticosterone or express DAX-1 (33, 34). Recently, Ragazzon *et al*. (17, 35) established two mouse ATC cell lines, ATC1 and ATC7. These are the first adrenocortical cell lines to possess a complete ZF cell phenotype and to produce corticosterone, and they are highly responsive to ACTH. However, studies using these cells have focused mainly on the cellular steroidogenic activation in response to sustained ACTH incubations. Because of our interest in understanding the mechanisms regulating dynamic transcription of steroidogenic genes in response to ACTH and stress (26), the aim of this investigation was to determine whether transcriptional activity in ATC cell lines would be activated by pulses of ACTH, and therefore these cells could be used to study steroidogenic signaling dynamics. Exploiting our previous *in vivo* observations that the genomic steroidogenic pathway is dynamically regulated in response to ultradian pulses of ACTH (26), we hypothesized that “pulses” of ACTH would also elicit pulsatile regulation of transcription in the ATC cells. To test this hypothesis, we treated ATC1 and ATC7 cells with 10 nM ACTH, either in the form of pulses or as constant incubation, and measured the time-dependent changes in transcription of ACTH-regulated steroidogenic related genes.

## Materials and Methods

### Cell culture

ATC1 and ATC7 mouse adrenal ZF cell lines were a kind gift from Dr. Pierre Val (Université Clermont Auvergne, Clermont-Ferrant, France). Both lines were originally established from separate adrenal tumors of transgenic mice (line 1, 4-month-old female and line 7, 7-month-old male) harboring the large T-antigen of simian virus 40 under the control of the adrenocortical-specific promoter of the Akr1b7 gene (35, 36). Cells were cultured on poly-l-lysine–coated 75 cm^2^ tissue culture flasks in a 1:1 mixture of DMEM and Ham’s F-12 medium (DMEM-F12) at 37°C in a 5% CO_2_/95% air atmosphere. The medium was supplemented with insulin (10 mg/mL), transferrin (5.5 mg/mL), and selenium (5 ng/mL), l-glutamine (2 mM), penicillin (100 U/mL), streptomycin (100 µg/mL), 2.5% horse serum, and 2.5% fetal bovine serum. The cells were passaged every 3 to 4 days and culture medium changed every 2 to 3 days. For all ACTH experiments, cells were seeded 2 to 3 days before experimentation into poly-l-lysine–coated six-well plates at 5 to 7 × 10^5^ cells per well. Cells were serum-starved in serum-free medium supplemented with 0.1% BSA ~16 hours before each experiment began.

### ACTH experiments

Porcine ACTH (A6303; Sigma, Gillingham, United Kingdom) was diluted to a final concentration of 1 mM in 0.01 M HCl/0.1% BSA, and stock aliquots were stored at −80°C. Before the experiment, 1 mM stock was diluted in serum-free media to make a working stock of either 10 µM or 1 µM. We added 20 µL of 10 µM or 1 µM working stock to the experimental well to produce a final concentration of ACTH of 100 nM or 10 nM, respectively. The choice of the 10 nM dose used in all experiments was based on Ragazzon *et al.* (17), and preliminary data based on this dosage in our experiments showed a similar induction of steroidogenic genes as found *in vivo* with a low-dose ACTH injection (10 ng per rat, IV) (26).

#### Pulsatile vs constant ACTH experiments

**Figure 1.**
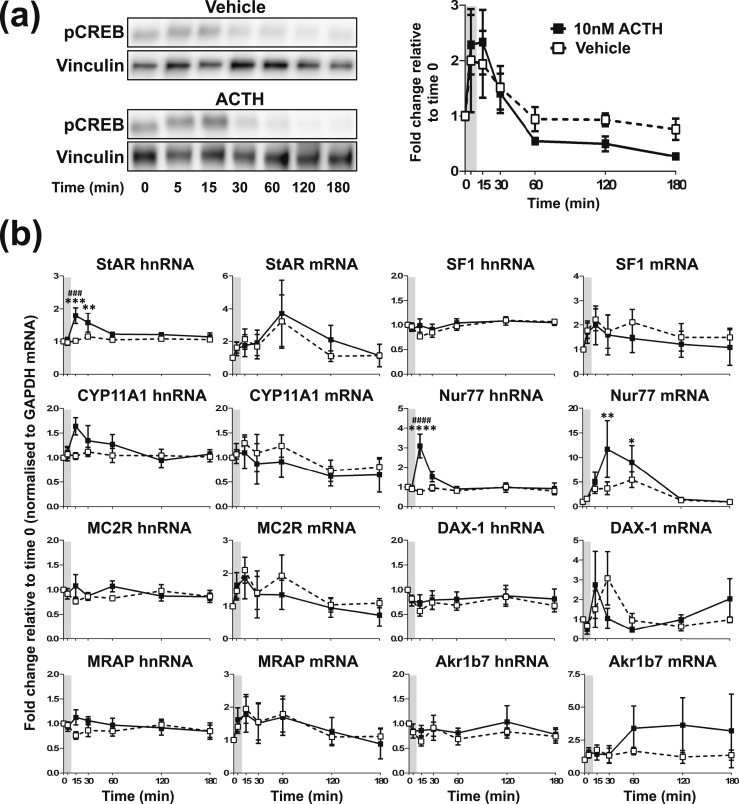
Effects of one pulse of 10 nM ACTH on steroidogenic signaling in ATC1 cells. Graph and representative Western blot demonstrating the effects of one pulse of ACTH or vehicle on (a) pCREB and (b) transcription of steroidogenic-related genes in ATC1 cells. Data shown are mean ± SEM of four separate experiments and are expressed as fold induction of untreated cells at time 0 minutes. Vertical gray bars represent an ACTH pulse. *P < 0.05, **P < 0.01, ***P < 0.001, and ****P < 0.0001 denote significant differences compared with time 0, analyzed by two-way ANOVA and Dunnett multiple comparisons test. ^###^*P* < 0.001 and ^####^*P* < 0.0001 denote significant differences between ACTH and vehicle at the respective time points, analyzed by two-way ANOVA and Sidak multiple comparisons test.

**Figure 2.**
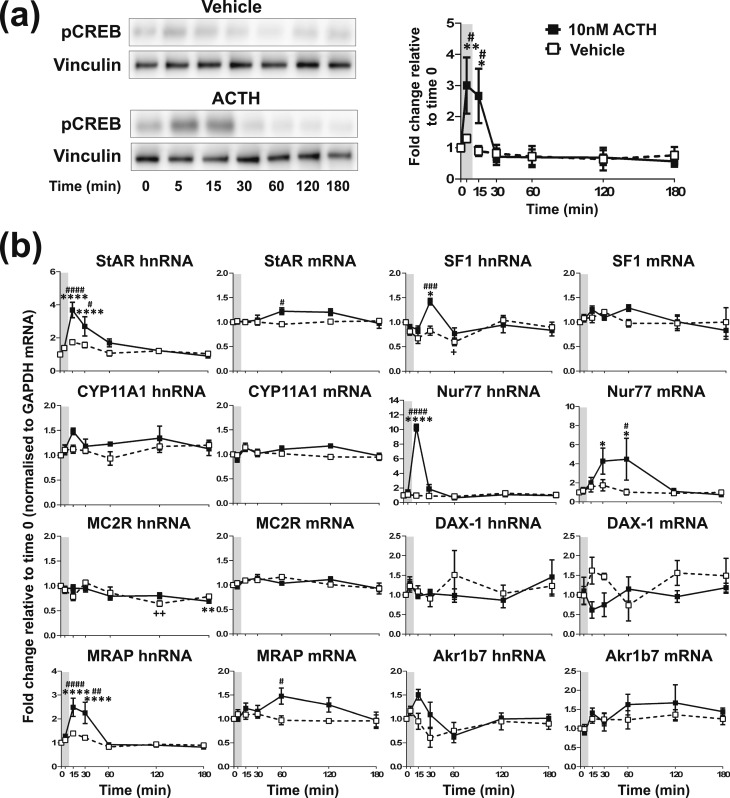
Effects of one pulse of 10 nM ACTH on steroidogenic signaling in ATC7 cells. Graph and representative Western blot demonstrating the effects of one pulse of ACTH or vehicle on (a) pCREB and (b) trancription of steroidogenic-related genes in ATC7 cells. Data shown are mean ± SEM of four separate experiments and are expressed as fold induction of untreated cells at time 0. Vertical gray bars represent an ACTH pulse. *P < 0.05, **P < 0.01, and ****P < 0.0001 denote significant effects of ACTH compared with time 0 minutes, analyzed by two-way ANOVA and Dunnett multiple comparisons test. ^#^*P* < 0.05, ^###^*P* < 0.001, and ^####^*P* < 0.0001 denote significant differences between ACTH and vehicle at the respective time points, analyzed by two-way ANOVA and Sidak multiple comparisons test.

**Figure 3.**
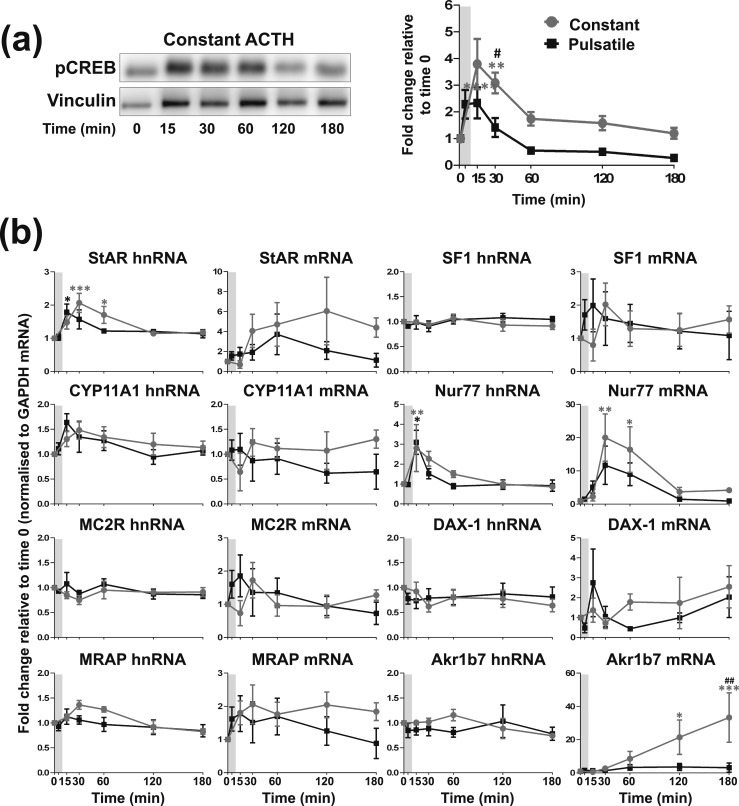
Effects of constant *vs* one pulse of ACTH on steroidogenic signaling in ATC1 cells. Graph and representative Western blot demonstrating the effects of constant 10 nM ACTH *vs* one pulse of 10 nM ACTH on (a) pCREB and (b) the transcription of steroidogenic-related genes in ATC1 cells. Because constant ACTH experiments were performed at the same time as the pulse experiments shown in Fig. 1, the pulse experiment data are the same as shown in Fig. 1, and only statistics for the constant treatment experiment are shown here. Data shown are mean ± SEM of four separate experiments and are expressed as fold induction of untreated cells at time 0. Vertical gray bars represent an ACTH pulse. *P < 0.05, **P < 0.01, ***P < 0.001, and ****P < 0.0001 denote significant differences compared with time 0 minutes, analyzed by two-way ANOVA and Dunnett multiple comparisons test. ^#^*P* < 0.05 and ^##^*P* < 0.01 denote significant differences between ACTH and vehicle at the respective time points, analyzed by two-way ANOVA and Sidak multiple comparisons test.

**Figure 4.**
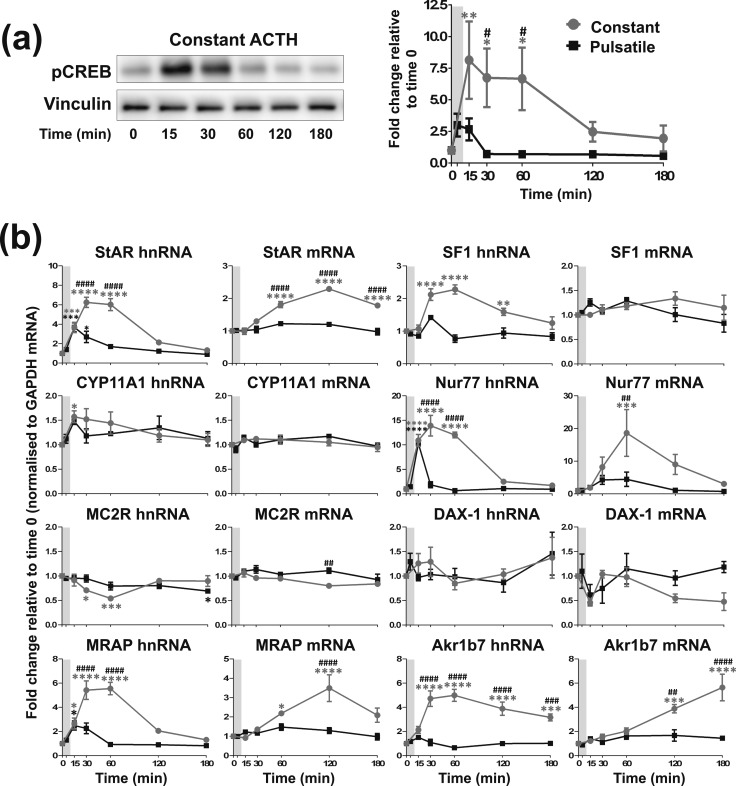
Effects of constant *vs* one pulse of ACTH on steroidogenic signaling in ATC7 cells. Graph and representative Western blot demonstrating the effects of constant 10 nM ACTH *vs* one pulse of 10 nM ACTH on (a) pCREB and (b) the transcription of steroidogenic-related genes. Because constant ACTH experiments were performed at the same time as the pulse experiments shown in Fig. 2, the pulse experiment data are the same as shown in Fig. 2, and only statistics for the constant treatment experiment are shown here. Data shown are mean ± SEM of four separate experiments and are expressed as fold induction of untreated time 0 minutes. Vertical gray bars represent an ACTH pulse. *P < 0.05, **P < 0.01, ***P < 0.001, and ****P < 0.0001 denote significant differences compared with time 0 minutes, analyzed by two-way ANOVA and Dunnett multiple comparisons test. ^#^*P* < 0.05, ^##^*P* < 0.01, ^###^*P* < 0.001, and ^####^*P* < 0.0001 denote significant differences between ACTH and vehicle at the respective time points, analyzed by two-way ANOVA and Sidak multiple comparisons test.

For the administration of 1 × 10 minute ACTH pulse, concentrated ACTH (1 µM) or vehicle (0.01 M HCl containing 0.1% BSA) was added to the well at time 0 minutes and left for 10 minutes. Media was removed by aspiration, and cells were washed with excess 1 × PBS. Cells were incubated in fresh serum-free media for the remaining time. For constant treatment, ACTH or vehicle was added to the well at time 0 minutes. Cells treated with either pulsatile or constant ACTH were then processed at specific time points, as indicated in Figs. 1–4.

#### Multiple ACTH pulse experiments

**Figure 5.**
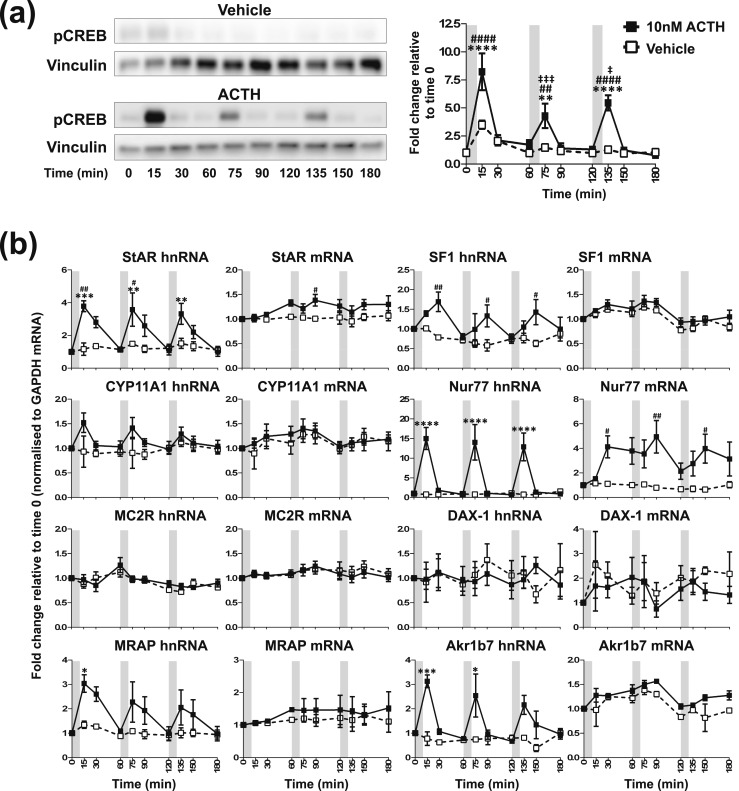
Multiple pulses of ACTH induce pulsatile steroidogenic signaling in ATC7 cells. Graph and representative Western blot depicting the dynamic effects of 3 × 10-minute ACTH pulses compared with 3 × 10-minute pulses of vehicle on (a) pCREB and (b) the transcription of steroidogenic-related genes. Data shown are mean ± SEM of four separate experiments and are expressed as fold induction of untreated cells at time 0 minutes. Gray bars represent ACTH pulses. *P < 0.05, **P < 0.01, ***P < 0.001, and ****P < 0.0001 denote significant differences compared with time 0 minutes, analyzed by two-way ANOVA and Tukey multiple comparisons test. The same tests were used to calculate the difference between the first and the following peaks: ^‡^*P* < 0.05 and ^‡‡‡^*P* < 0.001 represent significant differences compared with the first peak. ^#^*P* < 0.05, ^##^*P* < 0.01, and ^####^*P* < 0.0001 denote significant differences between ACTH and vehicle at the respective time points, analyzed by two-way ANOVA and Sidak multiple comparisons test.

Time concentrated ACTH (1 µM) was added to the well at time 0, 60, and 120 minutes; cells then were washed with excess 1 × PBS and incubated in fresh serum-free media at time 10, 70, and 130 minutes. Cells were then processed at specific time points as indicated in Fig. 5.

#### Pulsed experiments followed by 100 nM ACTH

In this experiment, the cells were “constant-ACTH-pulsed” (as opposed to one constant ACTH application from time 0) to control for the fresh 10 nM ACTH that was added to the media every hour in the ACTH-pulsed wells. For vehicle-pulsed, ACTH-pulsed, or constant-ACTH-pulsed, concentrated vehicle or ACTH (1 µM) was added to wells at time 0, 60, and 120 minutes, media was removed by aspiration at time 10, 70, or 130 minutes, and cells were washed with 1 × PBS and incubated either in serum-free media alone (vehicle-pulsed or ACTH-pulsed conditions) or in serum-free media containing 10 nM ACTH (constant-ACTH-pulsed) for 50 minutes. At 180 minutes, media was aspirated and replaced with serum-free media containing 100 nM ACTH.

### Quantitative RT-PCR

For RNA quantification, cells were lysed in RNA lysis buffer, and total RNA was purified with Ambion Pure-Link kit (Invitrogen, Thermo Fisher Scientific, Waltham, MA). The cDNA template was reverse transcribed from 1000 ng of total RNA with a Cloned AMV First-Strand cDNA synthesis kit (Invitrogen, ThermoFisher Scientific). Quantitative RT-PCR (qRT-PCR) was performed as previously described (37) with Power SYBR green PCR mix (Applied Biosystems, Thermo Fisher Scientific) and 4 ng cDNA template. qRT-PCR primers (listed in Table 1) were used at a final concentration of 200 nM and designed to span either an intronic-exonic region to detect levels of nascent transcript before splicing to mRNA [heteronuclear RNA (hnRNA)] or to span an exonic-exonic region to detect mature transcript (mRNA). The expression of each target gene was normalized to glyceraldehyde 3-phosphate dehydrogenase (GAPDH) mRNA as determined in a separate real-time PCR; relative hnRNA and mRNA levels were quantified with the 2^–∆∆CT^.

**Table 1.** qRT-PCR Primer Sequences

Gene	Transcript	Forward	Reverse
*StAR*	*hnRNA*	CTGTGCTCAGGATCCCAGTG	TGCAGGTCAATGTGGTGGAC
	*mRNA*	TCGTGAGCGTGCGCTGTACC	CTTCGGCAGCCACCCCTTCAG
*CYP11A1*	*hnRNA*	CTCAACCTGCCTCCAGACTTC	CCCTCCATGGTAGATTAGTGGC
	*mRNA*	CGCATCAAGCAGCAAAATTC	ATGCGCTCCCCAAATATAAC
*MC2R*	*hnRNA*	TCTGTTTAACCTCAGATCCTTCCAC	CTGGCCGTTAAGACGGGG
	*mRNA*	CCAAGGCCCTTCTAAGCCAG	CTTGCGGTGTCATTGGTGTG
*MRAP*	*hnRNA*	CAGCTGTGGGTGCGAGCCTC	CCCCAGCCTCTGCCTGGTCA
	*mRNA*	AGTCATGGCCAACGGGACCG	GGGACTGTGCCTCATCTGTGGGG
*SF1*	*hnRNA*	CATTATTCTTCCCTACAGGTGGCT	CGTACTGGACCTGGCGGTAG
	*mRNA*	AGGAGGAAAGGACGATCGGA	ACCTTGTCACCACACATGG
*Nur77*	*hnRNA*	ATGCAGCTTGTGTAGGCTTT	CTGCCCACTTTCGGATAACG
	*mRNA*	GCACAGCTTGGGTGTTGATG	CAGACGTGACAGGCAGCTG
*DAX-1*	*hnRNA*	AAAGATCCTGTGGTGAGCTGTTTTA	TAAGGATCTGCTGGGTTCTTCA
	*mRNA*	ACCGTGCTCTTTAACCCAGA	CCGGATGTGCTCAGTAAGG
*Akr1b7*	*hnRNA*	AGAATGCTGTGAAGCGGGAG	AGCTCTCAAACCTGCCCCTA
	*mRNA*	AGCTCAGGTTCTGATTCGGT	TCTCATCAAGCAAGTGGACCTC
*GAPDH*	*mRNA*	CCATCACTGCCACCCAGAAGA	GACACATTGGGGGTAGGAACA

### Western blot analysis

For protein quantification, cells were lysed in SDS lysis buffer (2% SDS, 50 mM Tris pH 6.8, 10% glycerol), and Western immunoblotting was performed as described in (26). In brief, all membranes were blocked with 1% BSA in Tris-buffered saline/0.05% Tween 20 and probed with a primary rabbit antibody directed to phosphorylated CREB (Ser133) [1:1000, #87G3; Cell Signaling Technologies, Danvers, MA; RRID: AB_2561044 (38)], followed by horseradish peroxidase–conjugated donkey *α*-rabbit secondary antibody [1:5000; Santa Cruz Biotechnology, Dallas, TX; RRID: AB_631745 (39)]. Blots were normalized to vinculin, detected with a goat *α*-vinculin [1:5000; Santa Cruz Biotechnology; RRID: AB_2272812 (40)] and donkey *α*-goat secondary antibody [1:5000; Santa Cruz Biotechnology; RRID: AB_641200 (41)]. The intensity of the protein target bands integrated with the area was quantified with a computer image analysis system, Image J (developed at the National Institutes of Health and freely available at http://rsb.info.nih.gov).

### cAMP quantification

Cellular cAMP levels were measured in whole cell lysate via a commercially available cAMP direct immunoassay (Abcam, Cambridge, United Kingdom) according to the manufacturer’s instructions.

### Corticosterone assay

Corticosterone was measured in triplicate in 100 μL media by RIA as previously described (26), with a specific rabbit anticorticosterone polyclonal antibody [RRID: AB_2762849 (42)] developed by professor Gabor Makara (Institute of Experimental Medicine, Budapest, Hungary) and kindly donated to us by Dr Dóra Zelena (Institute of Experimental Medicine, Budapest, Hungary). [125I]-Corticosterone was used as tracer (Institute of Isotopes, Budapest, Hungary). The interassay and intra-assay coefficients of variation of the corticosterone assay were 16.7% and 13.3%, respectively.

### Statistical analyses

All data are expressed as the mean ± SEM of values obtained from a minimum of three independent cell experiments. Data were analyzed via one-way ANOVA or two-way ANOVA; for clarity, all ANOVA results are reported in an online repository (43). Where appropriate, ANOVA was followed by a Dunnet, Sidak, or Tukey multiple comparisons test, as indicated in each figure legend. *P* values <0.05 were considered significant.

## Results

### One pulse of ACTH induces transient phosphorylation of CREB and dynamic transcription of steroidogenic genes in ATC1 and ATC7 cells

In this experiment, cells were treated with 10 nM ACTH for 10 minutes, and CREB phosphorylation (pCREB) and transcription of ACTH-regulated genes relating to the steroidogenic pathway were measured. In ATC1 cells [Fig. 1; statistical analyses are summarized in an online repository (43)], a pulse of ACTH prompted a transient but not significant increase in pCREB at 15 minutes [*P* = 0.1048; Fig. 1(a)] that was also echoed in the vehicle group (*P* = 0.3838). However, we did observe a transient increase in StAR hnRNA and Nur77 hnRNA in response to a pulse of ACTH, both with a peak of increase at 15 minutes (*P* = 0.0002 and *P* < 0.0001, respectively), which was not observed in the vehicle-treated group [Fig. 1(b)]. Regarding mature transcript (mRNA), a pulse of ACTH significantly increased the accumulation of Nur77 mRNA, with a peak in increase at 60 minutes (*P* = 0.0369). Neither treatment had any significant effect on the expression of the other genes investigated.

In ATC7 cells [Fig. 2; statistical analyses summarized in an online repository (43)], there was a rapid and transient increase in pCREB in response to a pulse of 10 nM ACTH, with a peak at 5 minutes [*P* = 0.0071; Fig. 2(a)] and a return to baseline by 30 minutes. In contrast to ATC1 cells, administration of a pulse of vehicle had no significant effect on pCREB (*P* = 0.9888 at 5 minutes). The rapid increase in pCREB induced by a pulse of ACTH was followed by a dynamic increase in StAR, MRAP, and Nur77 hnRNA, all with a peak at 15 minutes (*P* < 0.0001), and a dynamic increase in SF-1 hnRNA, with peak at 30 minutes [*P* = 0.0139; Fig. 2(b)]. Interestingly, the same treatment significantly decreased MC2R hnRNA at 180 minutes (*P* = 0.0076). Vehicle administration did alter the expression of SF-1 and MC2R hnRNA transcription with a significant reduction of SF-1 hnRNA at 60 minutes (*P* = 0.0159) and MC2R hnRNA at 120 minutes (*P* = 0.0018). Consistent with its effects on hnRNA, a pulse of ACTH also caused an increase in StAR, MRAP, and Nur77 mRNA (*P* = 0.0159, *P* = 0.0155, and *P* = 0.0155, respectively), all with a peak of expression at 60 minutes, but had no effect on the mRNA expression of the other genes investigated. In addition, there was no effect of a pulse of vehicle on mRNA expression for any of the genes investigated.

### Constant ACTH stimulates larger increases in pCREB and steroidogenic gene transcription in ATC1 and ATC7

Alongside the pulse experiments we also investigated the effects of constant 10 nM ACTH on the steroidogenic response in ATC1 [Fig. 3; statistical analyses summarized in an online repository (43)] and ATC7 [Fig. 4; statistical analyses summarized in an online repository (43)] cells and compared them with the responses observed after a pulse of ACTH (same data as shown in Figs. 1 and 2). In ATC1 cells (Fig. 3), constant incubation with ACTH resulted in a larger and longer pCREB induction than that observed with a pulse of ACTH, with a peak at 15 minutes [*P* < 0.0001; Fig. 3(a)]. Constant ACTH also produced a more pronounced induction in StAR hnRNA expression (peak at 30 minutes; *P* = 0.0007) and Nur77 mRNA expression (peak at 30 minutes; *P* = 0.0034) when compared with a pulse of ACTH [Fig. 3(b)]. In addition, constant ACTH treatment dramatically increased Akr1b7 mRNA expression over the 180-minute experiment (peaking at 180 minutes; *P* = 0.0004), as opposed to a pulse of 10 nM ACTH, which had no effect on Akr1b7 mRNA expression.

Similarly, for ATC7 cells (Fig. 4), constant 10 nM ACTH treatment induced a significant increase in pCREB expression that was higher and more prolonged than the effect observed after a pulse of ACTH, with a maximal response at 15 minutes [*P* = 0.0062; Fig. 4(a)] that remained significantly elevated at 30 and 60 minutes (*P* = 0.0329 and *P* = 0.0353, respectively). In addition to the differential effects on pCREB, there was a substantial difference in the effects of constant *vs* pulsatile ACTH treatment on gene transcription and mRNA accumulation [Fig. 4(b)]. Indeed, higher and more prolonged changes in hnRNA expression in response to constant ACTH treatment were observed in StAR hnRNA (peak at 15 minutes; *P* < 0.0001), Nur77 hnRNA (peak at 30 minutes; *P* < 0.0001), and MRAP, SF-1, and Akr1b7 hnRNA (peak at time 60 minutes; *P* < 0.0001). Conversely, constant ACTH led to a greater reduction in MC2R hnRNA expression when compared with a pulse of ACTH (lowest level at 60 minutes; *P* = 0.0002). In terms of mature transcript, constant ACTH administration leads to a far greater increase in the expression of StAR, MRAP, and Nur77 (StAR and MRAP mRNA, peak at 120 minutes, *P* < 0.0001; Nur77 mRNA, peak at 60 minutes, *P* = 0.0037) and Akr1b7 mRNA (peak at 180 minutes, *P* < 0.0001) when compared with one pulse of ACTH. Constant ACTH led to a reduction in MC2R mRNA expression when compared with a pulse of ACTH (*P* = 0.0018 at lowest level at 120 minutes). There was no significant difference between the two treatments for any of the other genes analyzed.

### Multiple pulses of ACTH induce pulsatile pCREB and steroidogenic gene transcription

Because adrenal ZF cells are exposed to trains of ultradian pulses of ACTH throughout the day in both humans and rodents (20, 25), we next studied the effects of multiple hourly pulses of ACTH on pCREB and steroidogenic transcriptional output. Because a pulse of vehicle increased CREB activity in ATC1 cells [Fig. 1(a)], we decided to continue our experiments on ATC7 cells only. Treatment with 3 × hourly 10-minute pulses of 10 nM ACTH resulted in concomitant hourly pulses of significant CREB activation [Fig. 5(a)]. The first pulse of ACTH induced a large pCREB response that peaked at 15 minutes (*P* < 0.0001). The second and third pulses of ACTH induced a significant increase in pCREB that peaked at 75 minutes (*P* = 0.0043) and 135 minutes (*P* = 0.0043), respectively, which was a significantly smaller pCREB response than the first pulse at 15 minutes (*P* = 0.0002 and *P* = 0.0392, compared with the first pulse at 15 minutes, respectively). Although 3 × hourly pulses of vehicle did not induce significant increases in pCREB [statistical analyses summarized in an online repository (43)], there was a noticeable induction after the first pulse (*P* = 0.1037 at 15 minutes) that did not occur after the second and third pulses of vehicle (*P* > 0.9999 at 75 and 135 minutes).

Administration of three pulses of ACTH also resulted in pulses of gene transcription [Fig. 5(b); statistical analyses summarized in an online repository (43)]. Each 10-minute ACTH pulse induced a significant increase in StAR and Nur77 hnRNA expression, with three peaks occurring (as observed for pCREB) at 15, 75, and 135 minutes (StAR hnRNA, *P* = 0.0005, *P* = 0.0019, and *P* = 0.0086, respectively; Nur77 hnRNA, all *P*s < 0.0001). Interestingly, in contrast to the pCREB response, all three ACTH pulses generated similar levels of pulsatile StAR and Nur77 hnRNA transcription (*i.e.,* no differences between the increases at 15, 75, and 135 minutes). The first pulse of ACTH also generated a pulse of MRAP hnRNA transcription, which peaked at 15 minutes (*P* = 0.0170), and although an increase in MRAP hnRNA was also observed for the second and third pulses, these effects were not significant. Three hourly pulses of 10 nM ACTH led to a pulsatile increase in SF-1 hnRNA expression, with the peaks occurring at 30, 90, and 150 minutes (*P* = 0.0059, *P* = 0.0384, and *P* = 0.0229, respectively). Finally, pulsatile ACTH incubation significantly increased Akr1b7 hnRNA at 15 and 75 minutes (*P* = 0.0004 and *P* = 0.0416), whereas the increase at 135 minutes induced by third pulse of ACTH was not significantly different when compared with time 0 (*P* = 0.3527). Regarding mRNA expression, three pulses of ACTH did not generate pulsatile changes in mRNA expression for any of the genes investigated but led to an accumulation in StAR mRNA (effect of treatment, *P* < 0.0001) and Nur77 mRNA (effect of treatment, *P* < 0.0001) over the 3-hour experiment.

### Adrenal ZF cells maintain responsiveness to ACTH over multiple ACTH pulses but not constant ACTH

**Figure 6.**
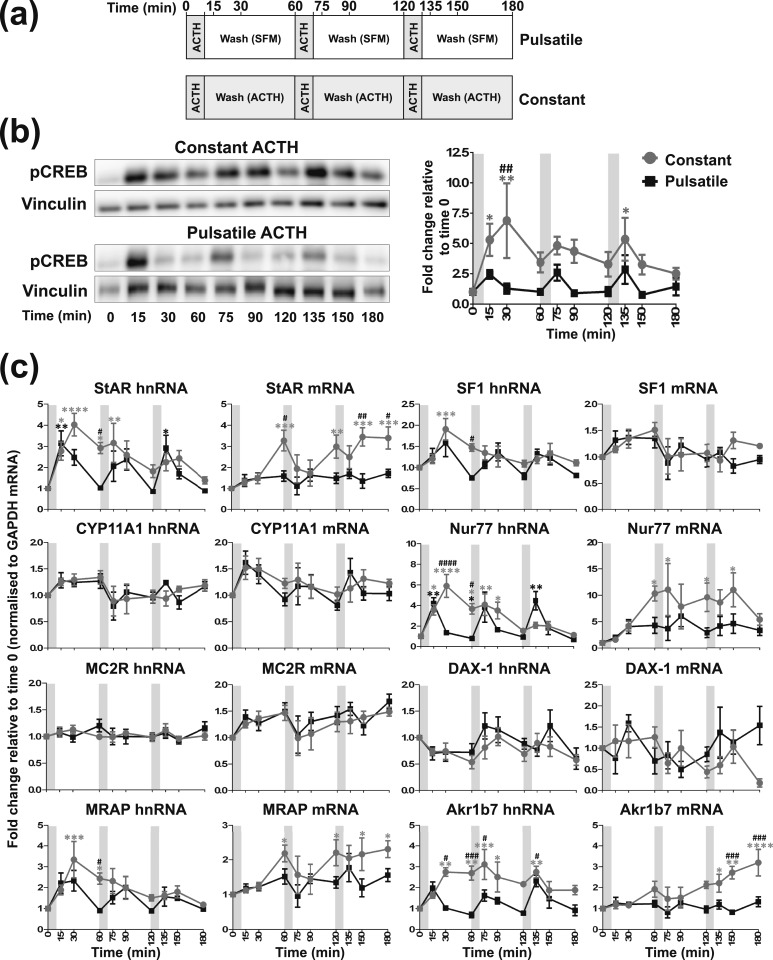
Effects of 3 × 10-minute constant pulses of ACTH vs 3 × 10-minute pulses of ACTH on steroidogenic signaling in ATC7 cells. (a) Schematic of experimental design. 20 µL of 1 µM ACTH (or equivalent vehicle) was added to wells at time 0, 60, and 120 minutes, and cells were washed with 1 × PBS media at time 10, 70, or 130 minutes. During the washes, cells were incubated in either serum-free media alone (vehicle-pulsed or ACTH-pulsed conditions) or in serum-free media containing 10 nM ACTH (constant-ACTH-pulsed) for 50 minutes. (b, c) Graphs and representative Western blots demonstrating the effects of hourly 10-minute vehicle pulses, hourly 10-minute 10 nM ACTH pulses, or constant 10 nM ACTH treatment on (b) pCREB and (c) steroidogenic-related gene transcription. Data shown are mean ± SEM of four separate experiments and are expressed as fold induction of untreated cells at time 0 minutes. Vertical gray bars represent ACTH pulses. *P < 0.05, **P < 0.01, ***P < 0.001, and ****P < 0.0001 denote significant differences compared with time 0 minutes analyzed by two-way ANOVA and Dunnett multiple comparisons test. ^#^*P* < 0.05, ^##^*P* < 0.01, ^###^*P* < 0.001, and ^####^*P* < 0.0001 denote significant differences between constant ACTH pulses and ACTH pulses at the respective time points, analyzed by two-way ANOVA and Sidak multiple comparisons test.

**Figure 7.**
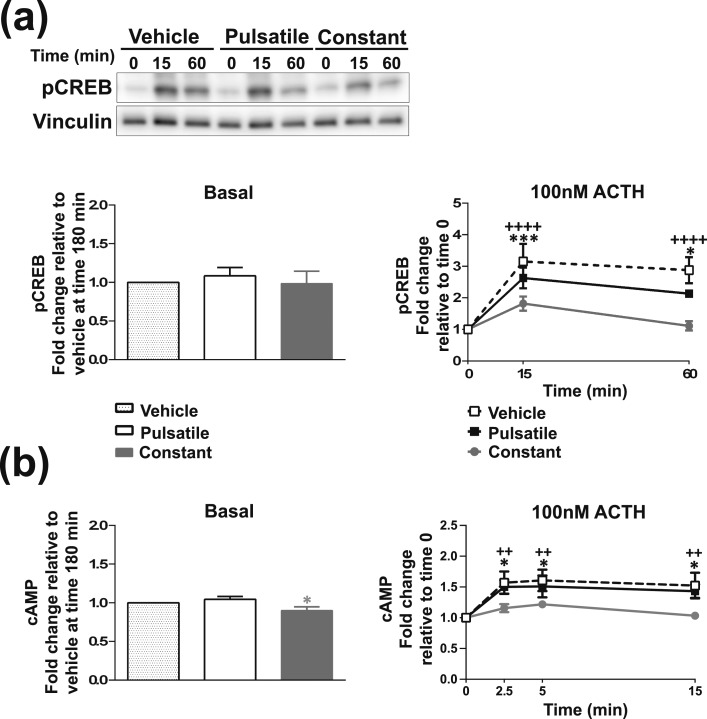
Effects of 3 × 10-minute constant pulses of ACTH vs 3 × 10-minute pulses of ACTH on pCREB and cAMP response to a large dose of ACTH in ATC7. (a) Representative Western blot and graphs showing pCREB levels at baseline 180 minutes (left panel) and the time-course effects of 100 nM ACTH (right panel) in vehicle-pulsed, constant ACTH, and ACTH-pulsed cells. (b) Graphs showing cAMP levels at baseline 180 minutes (left panel) and the time course effects of 100 nM ACTH (right panel) in vehicle-pulsed, constant ACTH, and ACTH-pulsed cells. Data in the left panels are mean ± SEM of four separate experiments and are expressed as fold induction of vehicle at time 0 minutes; data were analyzed by one-way ANOVA and Tukey multiple comparisons test: *P < 0.05 denotes a significant difference *vs* vehicle. Time-course data in the right panels are expressed as fold induction from the time 0 minutes (baseline180 minutes) of each respective group. Data were analyzed by two-way ANOVA and Dunnett or Sidak multiple comparisons test. *P < 0.05 and ***P < 0.001 denote significant differences compared with time 0 minutes (Dunnett test). ^++^*P* < 0.01 and ^++++^*P* < 0.0001 denote significant differences between constant-ACTH and ACTH-pulse treatment.

**Figure 8.**
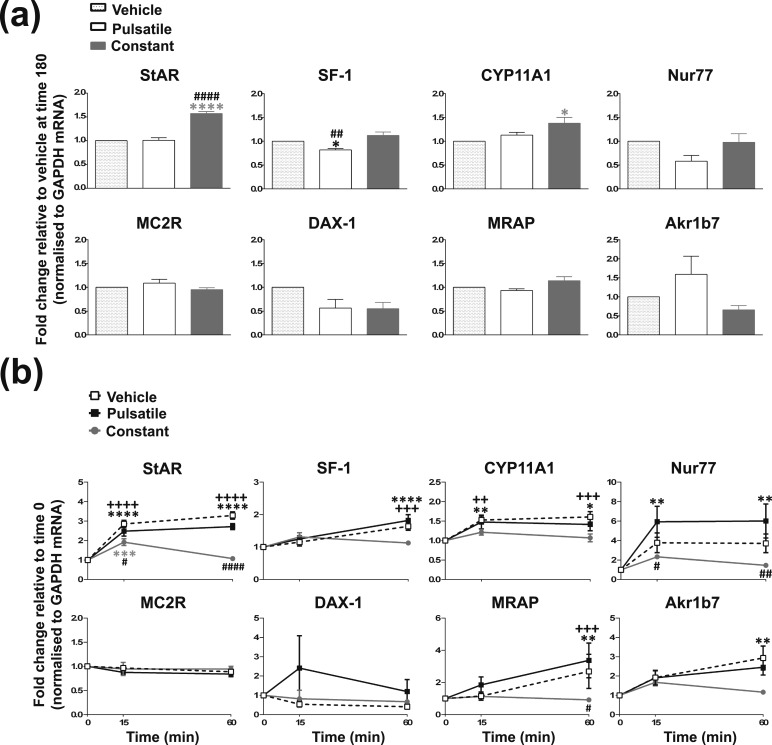
Effects of 3 × 10-minute constant pulses of ACTH vs 3 × 10-minute pulses of ACTH on steroidogenic gene transcription in response to a large dose of ACTH in ATC7. (a) Graphs illustrating steroidogenic gene transcription (hnRNA) at baseline 180 minutes in vehicle-pulsed, constant ACTH, and ACTH-pulsed cells. Data are mean ± SEM of four separate experiments and are expressed as fold induction of vehicle; data were analyzed by one-way ANOVA and Tukey multiple comparisons test: *P < 0.05 and ****P < 0.0001 denote significant differences *vs* vehicle. ^##^*P* < 0.01 and ^####^*P* < 0.0001 denote significant differences between constant ACTH and ACTH-pulsed treatment. (b) Time course effects of 100 nM ACTH steroidogenic gene transcription (hnRNA) in vehicle-pulsed, constant ACTH, and ACTH-pulsed cells. Data shown are mean ± SEM of four separate experiments and are expressed as fold induction from the time 0 minutes (baseline 180 minutes) of each respective group. **P < 0.01, ***P < 0.001, and ****P < 0.0001 denote significant differences compared with each group at time 0 minutes, analyzed by two-way ANOVA and Dunnett multiple comparisons test. ^#^*P* < 0.05, ^##^*P* < 0.01, and ^####^*P* < 0.0001 denote significant differences between constant ACTH pulses and ACTH pulses at the respective time points, and ^++^*P* < 0.01 and ^+++^*P* < 0.001 denote significant difference between constant ACTH pulses and vehicle, analyzed by two-way ANOVA and Sidak multiple comparisons test.

We previously observed in the rat adrenal cortex that exposure to pulsatile ACTH, but not constant ACTH, is necessary for optimal hormonal secretion and genomic activation of the steroidogenic pathway (27). We therefore wanted to elucidate whether this also applied to ATC7 cells *in vitro*. To test our hypothesis, cells were exposed to either pulsatile-vehicle, pulsatile-ACTH, or constant-ACTH and then treated with a larger dose of ACTH (100 nM) for 1 hour (Figs. 6–8). As explained in the Materials and Methods section, in this experiment the constant-ACTH cells were “constant-ACTH-pulsed” (as opposed to one constant ACTH application from time 0) to control for the fresh 10 nM ACTH that was added to the media every hour in the ACTH-pulsed wells.

First, we assessed the effects of 3 × 10-minute constant-pulses of ACTH vs 3 × 10-minute pulses of ACTH on the steroidogenic signaling in ATC7 cells. A schematic of the experimental design is shown in Fig. 6(a) and statistical analyses of data are summarized in an online repository (43). Compared with 3 × 10-minute pulses of ACTH, 3 × 10-minute constant-pulses of ACTH resulted in a higher effects on pCREB [Fig. 6(b)], with a significant increase at 15, 30, and 135 minutes (*P* = 0.033, *P* = 0.0014, *P* = 0.029, respectively) compared with time 0, and higher levels at 30 minutes compared with 3 × 10-minute pulses of ACTH (*P* = 0.0031). The effects of 3 × 10-minute constant-pulses of ACTH vs 3 × 10-minute pulses of ACTH on genes transcription and mRNA accumulation were diverse [(Fig. 6(c)]. Levels of StAR hnRNA were elevated between 15 and 75 minutes (*P* < 0.05) in cells treated with constant ACTH, whereas the increase in mRNA was maintained throughout the treatment. Similarly, SF-1 hnRNA levels were significantly high only at 30 minutes (*P* = 0.0005), and no increase was detected in SF-1 mRNA. Furthermore, Nur77 and MRAP hnRNA levels were elevated only between 15 and 90 minutes (*P* < 0.05) and at 30 minutes (*P* = 0.0002) and 60 minutes (*P* = 0.0472), respectively, whereas mRNA levels for both genes were high throughout the treatment. In contrast, levels of Akr1b7 hnRNA were high throughout the treatment (30 to 135 minutes; *P* < 0.05), and mRNA levels increased toward the end of the treatment (135 to 180 minutes; *P* < 0.05). Significant differences were also observed when compared with cells treated with pulsatile ACTH, with higher StAR hnRNA at 60 minutes (*P* = 0.0321); higher StAR mRNA at 60, 150, and 180 minutes (*P* = 0.0313; *P* = 0.0032; *P* = 0.0325, respectively); higher Nur77 hnRNA at 30 and 60 minutes (*P* < 0.0001 and *P* = 0.0150, respectively); higher MRAP hnRNA at 30 minutes (*P* = 0.0393); higher Akr1b7 hnRNA at 30, 60, 75, and 120 minutes (*P* < 0.05); and higher Akr1b7 mRNA at 150 and 180 minutes (*P* < 0.001). It is noteworthy that, although the experimental design of the pulsatile ACTH incubation in this experiment is the same as in the experiment described above (data shown in Fig. 5) and the dynamic response to ACTH pulses in this experiment is maintained, the amplitude of increase of both pCREB and the hnRNA of some of the genes (including StAR, MRAP, and Akr1bt hnRNA) was lower than in the previous experiment, presumably because of different responsiveness of the cells at different passages. However, it is important to point out that this reduced responsiveness will presumably also apply to the cells incubated with vehicle or constant ACTH, and therefore a comparison between these groups is still reliable.

After exposure to pulsatile-vehicle, pulsatile-ACTH (3 × 10-minute pulses), or constant-ACTH (3 × 10-minute constant-pulse), cells were then treated with a larger dose of ACTH (100 nM) for 1 hour (Figs. 7 and 8). The levels of pCREB were not different between the three experimental groups before 100 nM of ACTH incubation [Fig. 7(a), left panel], whereas pCREB response to 100 nM ACTH was reduced in the constant ACTH-treated cells when compared with vehicle-pulsed (*P* = 0.0003 at 60 minutes) or ACTH-pulsed cells [*P* = 0.0389; Fig. 7(a), right panel] [statistical analyses summarized in an online repository (43)]. Specifically, 100 nM ACTH significantly increased pCREB levels in vehicle-pulsed cells at 15 and 60 minutes (*P* < 0.0001) and in ACTH-pulsed cells at 15 minutes (*P* = 0.0005) and 60 minutes (*P* = 0.0125); however, 100 nM ACTH had no significant effect on pCREB in the constant group at either time point (*P* = 0.0775 and *P* = 0.9422, respectively). To understand whether the reduction in CREB activity in response to constant ACTH was a result of inhibition of steroidogenic signaling upstream of CREB, we investigated the effects of pulsatile-vehicle, pulsatile-ACTH, or constant-ACTH on cAMP activity in response to 100 nM ACTH. As shown in Fig. 7(b), left panel, the levels of cAMP in the ACTH-pulsed and constant ACTH group were not significantly different from the vehicle-pulsed group before 100 nM ACTH incubation [statistical analyses summarized in an online repository (43)]. However, cAMP levels were significantly lower in the constant ACTH-treated cells when compared with the ACTH-pulsed cells (*P* = 0.0423). Addition of 100 nM ACTH significantly increased cAMP levels by 2.5 minutes in the vehicle-pulsed and the ACTH-pulsed cells [*P* < 0.01, *P* < 0.05, respectively; Fig. 7(b), right panel], which remained elevated at 15 minutes in both treatment groups (*P* < 0.01, *P* < 0.05, respectively). However, ACTH did not significantly increase cAMP levels in the constant ACTH-treated cells at 2.5 (*P* = 0.9981), 5 (*P* = 0.9716), or 15 minutes (*P* = 0.9999).

To assess whether the decrease in pCREB and cAMP activity in response to 100 nM ACTH was associated with a lower steroidogenic transcriptional activity in constant-ACTH (3 × 10-minute constant-pulse)-treated cells, we measured gene transcription across the three treatments groups (Fig. 8). Before 100 nM of ACTH incubation [Fig. 8(a)], StAR hnRNA expression levels were higher in constant-ACTH cells, compared with both vehicle-pulsed (*P* < 0.0001) and ACTH-pulsed (*P* < 0.0001) cells. CYP11A1 hnRNA was also significantly higher in constant-ACTH cells vs vehicle-treated cells (*P* = 0.0194), whereas SF-1 hnRNA expression levels were significantly lower in the ACTH-pulsed group vs vehicle (*P* = 0.0377) and constant-ACTH treated cells (*P* = 0.0019). There was no significant difference in hnRNA expression between treatment groups for Nur77, MC2R, MRAP, DAX1, and Akr1b7 hnRNA before incubation with 100 nM ACTH [statistical analyses summarized in an online repository (43)].

The effect of 100 nM ACTH on hnRNA expression is shown in Fig. 8(b) [statistical analyses summarized in an online repository (43)]. In vehicle-pulsed cells, 100 nM ACTH significantly increased StAR and CYP11A1 hnRNA at 15 minutes (StAR, *P* < 0.0001 and CYP11A1, *P* = 0.0030) and StAR, CYP11A1 MRAP, and SF-1 at 60 minutes (StAR, *P* < 0.0001; CYP11A1, *P* = 0.0008; MRAP, *P* = 0.0003; SF-1, *P* = 0.0002). There was a trend toward an increase in Nur77 hnRNA at 15 and 60 minutes and Akr1b7 hnRNA at 60 minutes, but it did not reach significance (*P* = 0.0844, *P* = 0.0668, respectively). As with vehicle-pulsed cells, 100 nM ACTH significantly increased the expression of StAR and CYP11A1 hnRNA in ACTH-pulsed cells at 15 minutes (StAR, *P* < 0.0001 and CYP11A1, *P* = 0.0070) and 60 minutes (StAR, *P* < 0.001 and CYP11A1, *P* = 0.0192) and MRAP and SF-1 hnRNA at 60 minutes (MRAP, *P* < 0.0850 and SF-1, *P* < 0.0001). In contrast to vehicle-pulsed cells, 100 nM ACTH also significantly increased Nur77 hnRNA at 15 minutes (*P* = 0.0015) and 60 minutes (*P* = 0.0013) and Akr1b7 hnRNA at 60 minutes (*P* = 0.0086). In keeping with the pCREB results, there was a reduction in the ATC7 cells transcriptional response to 100 nM ACTH in the constant-ACTH group. Of the genes investigated, StAR was the only steroidogenic gene to respond to 100 nM ACTH in the constant-ACTH treated group, with significantly higher hnRNA expression occurring at 15 minutes (*P* = 0.0003). In summary, the steroidogenic response to 100 nM ACTH is greatly reduced in cells that have been exposed to constant treatment of 10 nM ACTH as opposed to no ACTH treatment or pulsatile 10 nM ACTH treatment.

## Discussion

The overriding aim of this investigation was to determine whether ATC1 and ATC7 cells represent a valid and reliable model to study ACTH-mediated steroidogenic transcriptional dynamics *in vitro*. Here we show that hourly pulses of 10 nM ACTH administered to ATC7 cells can reliably stimulate a pulsatile pattern of steroidogenic transcriptional activity that is comparable to that observed in rat adrenals *in vivo*. We also provide further evidence that pulsatile, but not constant ACTH, is necessary for optimal activation of steroidogenesis in adrenal ZF cells.

Except for a few early studies on isolated adrenocortical cells (29, 30) in recent years there has been a lack of research into the effects of ACTH pulsing dynamics on steroidogenesis in adrenal cells *in vitro,* presumably because of the previous lack of viable ZF cell lines. The data we show here suggest that ATC1 and ATC7 cell lines, the first immortalized adrenocortical tumor cell lines to exhibit complete ZF cell phenotypes, can provide a good *in vitro* model to study steroidogenic transcriptional dynamics. Previously, we showed *in vivo* that an ultradian pulse of ACTH results in rapid and transient activation of the genomic steroidogenic pathway in the rat adrenal (26), that is, a rapid and transient activation of the steroidogenic-related proteins CREB and increases in the primary transcript (hnRNA) of the steroidogenic-related genes StAR, CYP11A1, SF-1, MC2R, MRAP, and Nur77. These changes in primary transcript were accompanied by a transient increase in MC2R, Nur77, and CYP11A1 mature transcript (mRNA) and a gradual increase in StAR and MRAP mRNA. In the same study we also observed a rapid and transient reduction in DAX-1 hnRNA, which is consistent with the inhibitory effect of ACTH on DAX-1 gene expression (17).

To replicate our *in vivo* findings by using an *in vitro* approach, we exposed both ATC cell lines to hourly pulses of 10 nM ACTH and measured the activation of the genomic steroidogenic pathway. We also measured the expression levels of the Akr1b7 (aldo-keto reductase family 1, member B7) gene, a ZF zonal marker and a highly ACTH-inducible gene that we used to corroborate ACTH responsiveness in these cells. As we found *in vivo,* a pulse of 10 nM ACTH provoked the rapid and transient activation of the genomic pathway in both ATC1 and ATC7 cells. However, of the two cell types, the ACTH-induced pattern of CREB activation and gene transcription in ATC7 cells more closely matched what we observed in the rat adrenal. For example, a 10-minute pulse of 10 nM ACTH induced a rapid and transient fourfold increase in StAR transcription in ATC7 cells [Fig. 2(c)] that followed the fourfold increase and pattern of StAR transcription in the rat adrenal in response to an ultradian pulse (26). The pattern of CYP11A1, MRAP, SF-1, and Nur77 gene transcription in ATC7 cells with a 10-minute pulse of 10 nM ACTH [Fig. 2(c)] also mirrored what we observed *in vivo* (26). In contrast to the robust dynamic modulation of the genomic steroidogenic pathway in ATC7 cells, a pulse of ACTH induced a significant pulse of transcription only of the steroidogenic genes StAR and Nur77 (and a slight induction of CYP11A1) in ATC1 cells [Fig. 1(c)].

When comparing the effects of a pulse of ACTH with that of constant ACTH, we found that 3 hours of constant incubation with 10 nM ACTH provoked an elevated and prolonged activation of the genomic steroidogenic pathway in ATC7 cells (Fig. 4). This finding closely resembles the exaggerated activation of the genomic steroidogenic pathway in the rat adrenal in response to a high dosage of ACTH (26). However, aside from a much larger accumulation of Akr1b7 mature transcript in response to constant 10 nM ACTH, the activation of the genomic steroidogenic pathway in ATC1 cells in response to either a pulse or constant 10 nM ACTH was similar. It is not clear why there are such differences in hormone sensitivity between the ATC1 and ATC7 cells, particularly because previous studies reported that ACTH-mediated steroidogenic-related gene expression is very similar in both cell lines (17, 35). However, these studies measured the transcriptional output of ATC cells in response to a larger dosage of ACTH (10 times higher) and over longer periods (≥3 hours). We have also investigated the transcript expression of an additional two highly ACTH-inducible steroidogenic-related genes, MRAP (44. 45) and Nur77 (46), which to our knowledge have not previously been measured in these cell types. Therefore, the lower the dosage of ACTH (10 nM), the shorter time course, and the measurement of two more highly inducible genes in this study may emphasize the differences in steroidogenic-related gene transcriptional output between the two cell types. Ragazzon *et al*. (17, 35) showed that the basal levels of corticosterone secretion were seven times higher in ATC7 cells than in ATC1 cells. Therefore, it is tempting to assume that the observed difference in sensitivity to ACTH may be attributed to different baseline levels of corticosterone. We attempted to measure corticosterone levels in our study, and, consistent with Ragazzon *et al.* (17), we found undetectable or low levels of hormone in unstimulated ATC1 cells (~2.3 ng/10^6^ cells) and higher levels of corticosterone in unstimulated ATC7 cells (~84.4 ng/10^6^ cells). However, in contrast to the study of Ragazzon *et al.* (17), we did not see any effect of ACTH treatments on corticosterone levels in either cell line. This discrepancy is probably caused by the differential methods of corticosterone assay (RIA in our study, mass spectrometry in the Ragazzon *et al*. (17) study). A body of literature supports the hypothesis that glucocorticoids have inhibitory actions on the steroidogenic pathway. For example, glucocorticoid pretreatment has been shown to inhibit ACTH-stimulated glucocorticoid synthesis (47, 48), repress StAR transcription (49), and increase the transcription of the corepressor DAX-1 (50). Therefore, we would expect a reduced level of transcription in response to ACTH in ATC7 cells when compared with ATC1 cells, yet we observed the opposite. Interestingly, it has also been reported that prolonged exposure to glucocorticoids can indeed increase both StAR transcription and cortisol production (51), and high levels of DAX-1 can increase steroidogenic gene expression (52).

Because the pattern of activation of the genomic steroidogenic pathway in response to a pulse of 10 nM ACTH in ATC7 cells, but not ATC1 cells, resembles the response to a physiological ACTH pulse *in vivo,* we believe that these cells would be the more appropriate cell type to use in studying adrenal transcriptional dynamics *in vitro*. This belief is supported by the nonspecific apparent effect of a pulse of vehicle on CREB phosphorylation in ATC1 cells [Fig. (b)]. The reason for this effect is unclear because the vehicle, HCl/BSA, is a routine reconstitution buffer used in tissue culture. Altering the pH conditions of tissue culture medium can have profound effects on cell biology (53), but it would be surprising if such a small amount of HCl added to the experimental well could affect pCREB levels, especially because ATC1 cells exposed to constant vehicle do not show this response (pCREB levels at 15 minutes, constant-vehicle, 1.2-fold ± 0.218 SEM increase vs pulse-vehicle 1.94 ± 0.6; Hazell, unpublished observations). This result suggests that it might instead be the fluid shear stress generated by the pulse washout at 10 minutes rather than the effects of vehicle itself administered at time 0 minutes that generated the increase in pCREB observed at 15 minutes. Mechanical stress such as fluid shear stress can disturb the membrane lipid bilayer and activate associated G proteins in a process probably driven by conformation changes in membrane receptors such as G protein-coupled receptors and transmembrane integrin receptors (54–56). This disruption can activate various transduction pathways, including the cAMP/PKA/CREB cascade (56, 57).

Regardless of the source, the nonspecific effects of a pulse of vehicle on pCREB levels did not affect transcription of the ACTH responsive genes StAR and Nur77 in ATC1 cells. This finding suggests that other factors that mediate the ACTH/pCREB-stimulated transcription of these genes [*e.g.,* the CREB coactivator transducer of CREB regulated activity, CRTC, as known as TORC (58–60)] may not be recruited or activated by the vehicle or mechanical stimulation. In contrast, there were only slight effects of a pulse of vehicle on gene transcription in ATC7 cells. A pulse of vehicle reduced MC2R and SF-1 gene expression, but the pattern of activation was different from that observed after ACTH. Therefore, it is possible that mechanical stimulation may induce or activate factors independent of the conical steroidogenic genomic pathway that interfere with the transcription of these steroidogenic-related genes.

The pulsatile secretion of ACTH suggests that adrenal steroidogenic transcriptional activity is also pulsatile throughout the day; therefore, we tested whether we could recreate such dynamic adrenal responses *in vitro*. We found that administration of three hourly 10 nM ACTH pulses to ATC7 cells resulted in three robust pulses transcriptional activation of the steroidogenic pathway. As previously seen in the rodent adrenal in response to physiological ACTH pulses (26–28, 60), we observed successive pulses of CREB activation and StAR, MRAP, SF-1, and Nur77 gene transcription (and a trend toward pulses of CYP11A1 gene transcription) but found no changes in MC2R and DAX-1 gene transcription. This is unsurprising considering that Ragazzon *et al.* (17) found that the MC2R gene is unresponsive to ACTH stimulation in ATC cells and that significant reductions in DAX-1 mRNA expression occur only after 3 to 6 hours of ACTH treatment (which is beyond the time points used in our study). In addition to the steroidogenic-related genes that we have previously investigated *in vivo,* we also measured transcription of the zonal marker Akr1b7. The Akr1b7 gene encodes aldose reductase–like protein, a detoxifying enzyme that removes harmful aldehydes generated during steroidogenesis (61). This study shows episodic regulation of Akr1b7 gene transcription in response to pulses of ACTH, which is not surprising considering that Akr1b7 gene expression is known to be upregulated via the canonical ACTH/cAMP/PKA/CREB pathway (17). Therefore, it is tempting to speculate that the coordinated dynamic upregulation of Akr1b7 transcription provides the cell with detoxifying enzymes that scavenge harmful byproducts produced by transient surges in steroidogenic activity in response to pulses of ACTH.

We have previously shown that pulses of ACTH are necessary for optimal activation of the adrenal genomic steroidogenic pathway *in vivo* (27). Here we confirm that pulses are necessary to maintain the transcriptional responsiveness of adrenal cells to ACTH, because we observed a large activation of the genomic steroidogenic pathway in response to a high dose of ACTH (100 nM) in ATC7 cells pretreated with hourly 10-minute ACTH-pulses but not in cells pretreated with constant-ACTH. This suggests that constant-ACTH desensitizes the genomic steroidogenic pathway in ATC7 cells to further stimulation by ACTH. The reduction in steroidogenic signaling begins upstream of CREB, because we found a decrease in cAMP levels in cells pretreated with constant-ACTH but not pulses of ACTH. This finding is consistent with other studies in which a reduction in adenylyl cyclase activity and cAMP levels in response to prolonged ACTH exposure was observed (62–64). However, rather than attributing the reduction in cAMP levels to a decrease in adenylyl cyclase activity or an increase in phosphodiesterase activity (62,63), these studies suggest that desensitization of the genomic steroidogenic pathway occurs as a result of a reduction in MC2R signaling due to ACTH mediated PKA-dependant receptor desensitization and G protein–coupled receptor kinase internalization via clathrin-coated vesicles (64, 65). Our observation that MC2R transcript expression remains unchanged after constant ACTH treatment (see Figs. 6–8) also supports the hypothesis that there is a desensitization or internalization of the MC2R in response to constant ACTH in adrenal cells. However, because of the current lack in viable MC2R antibodies, we were unable to confirm whether MC2R protein levels did indeed change. Alternatively, the decrease in MRAP mRNA in response to 100 nM ACTH in constant-ACTH treated cells when compared with pulsed-ACTH cells offers another mechanism for a reduction in MC2R signaling in ATC7 cells. Because MRAP is a single-transmembrane domain protein that promotes the trafficking of MC2R from the endoplasmic reticulum to the cell surface and renders MC2R responsive to ACTH (8, 66, 67), the decline in MRAP gene transcription may suggest that there is less MRAP protein available to support MC2R signaling in cells treated with constant ACTH. Clearly, additional studies are needed to determine the effects of ACTH pulsing dynamics on MC2R/MRAP signaling in ZF cells.

In summary, administration of hourly 10-minute pulses of 10 nM ACTH to ATC7 cells results in the pulsatile activation of the genomic steroidogenic pathway that resembles the physiological pattern of activation in the rodent. Therefore, our data support our hypothesis of the importance of intracellular dynamics in the regulation of steroidogenesis and provide strong evidence that the ACT7 cell line provides a good model for investigating the transcriptional effects of ultradian ACTH pulses *in vitro*. However, cell culture does not reproduce all the cell-cell communications found in intact tissues, and it is not surprising that the response of ATC7 to ACTH is not the exact replicate of what is observed *in vivo,* such as the lack of change in MC2R and DAX-1 gene transcription in response to ACTH pulses (in the investigated time frames). However, the ATC7 cells do offer an excellent model of adrenal regulation at the cellular level and allow detailed studies of the dynamic intracellular changes taking place during ACTH mediated transcriptional activation of steroidogenic pathways.

## Abbreviations:

CYP11A1: cytochrome P450 side chain cleavage enzyme
GAPDH: glyceraldehyde 3-phosphate dehydrogenase
hnRNA: heteronuclear RNA
HSL: hormone-sensitive lipase
pCREB: CREB phosphorylation
PKA: protein kinase A
qRT-PCR: quantitative RT-PCR
SF-1: steroidogenic factor-1
StAR: steroidogenic acute regulatory protein
ZF: *zona fasciculata*
